# External iliac artery thrombosis as a result of acetabular fixation through the ilioinguinal approach: a case report

**DOI:** 10.1097/MS9.0000000000001408

**Published:** 2023-10-17

**Authors:** Dorsa Hadavi, Parmida Shahbazi, Niloofar Gholami, Amirhossein Hajialigol, Salman Azarsina

**Affiliations:** a Radiology Department, Imam Khomeini Hospital Complex, Tehran University of Medical Sciences, Tehran, Iran; b Orthopaedic Department, Orthopaedic Subspecialty Research Center (OSRC), Sina University Hospital, Tehran University of Medical Sciences, Tehran, Iran; c Cardiovascular Research Center, Alborz University of Medical Sciences, Alborz, Iran; d Alborz Office of Universal Scientific Education and Research Network (USERN), Alborz University of Medical Sciences, Karaj, Iran; e Department of Orthopedic Surgery, Shahid Madani Hospital, Alborz University of Medical Sciences, Karaj, Iran

**Keywords:** acetabulum, case report, pelvic bones, thrombosis

## Abstract

**Introduction and importance:**

Acetabular fractures mostly occur in young people who are involved in high-energy trauma and they are treated by orthopedic trauma surgeons. Patients with acetabular fractures are at high risk for different kinds of complications. We report a case of postoperative thrombosis of the external iliac artery following fixation surgery performed by an ilioinguinal approach while receiving thromboprophylaxis during admission to the hospital.

**Case presentation:**

A 57-year-old healthy woman presented with a left both-column acetabular fracture and underwent acetabular fixation through the ilioinguinal approach. The patient was receiving antithrombotic prophylaxis medications in the course of treatment.

**Clinical discussion:**

During her convalescence, while at the hospital, she was diagnosed with left external iliac artery thrombosis, needing surgical thrombectomy. These severe and rare complications will lead to uncertainty about a commonly used ilioinguinal approach. Postoperative arterial thrombosis may be rare in patients undergoing acetabular fixation surgery but searching for signs and symptoms of this condition is always necessary.

**Conclusion:**

It is possible to prevent severe complications by performing a routine measurement of the distal arterial pressure after similar surgeries.

## Introduction

Highlights

- Rare postoperative left external iliac artery thrombosis in ilioinguinal approach for acetabular fixation despite prophylaxis.
- Importance of routine measurement of distal arterial pressure and vigilance for signs and symptoms.
- Emphasizes prevention measures to avoid severe complications in similar surgeries.

Acetabular fractures, occurring mostly in young individuals involved in high-energy trauma^1^, pose a significant challenge for orthopedic trauma surgeons, with an annual incidence rate of 3 per 100 000^2^.

The decision to pursue a surgical approach is not always a straightforward one, but many studies showed that surgical approaches in acetabular fractures had better results than nonoperative options^3^. Anatomical reduction is the most decisive factor for good long-term results^3,4^. The ilioinguinal approach is the preferred approach to access the site of the bone fixation, specifically fractures of the anterior wall of the acetabulum^5^.

Patients with acetabular fractures are at increased risk of various complications, with neurovascular and thromboembolic incidents being the most commonly encountered^6^. Critical limb ischemia resulting from arterial thrombosis following acetabular fixation surgeries poses a limb-threatening condition; therefore, prompt action is imperative to ensure timely diagnosis and treatment^7^. The precise incidence of postsurgical arterial thrombosis remains unknown due to collateral circulation that perfuses the extremities^8^.

To date, only two studies report the arterial thrombosis following acetabular fracture surgery conducted through the ilioinguinal approach^9,10^. We present a case of postoperative external iliac artery thrombosis after acetabular fixation surgery, conducted through an ilioinguinal approach, despite the patient receiving thromboprophylaxis during her hospital stay.

## Case presentation

**Figure 1:**
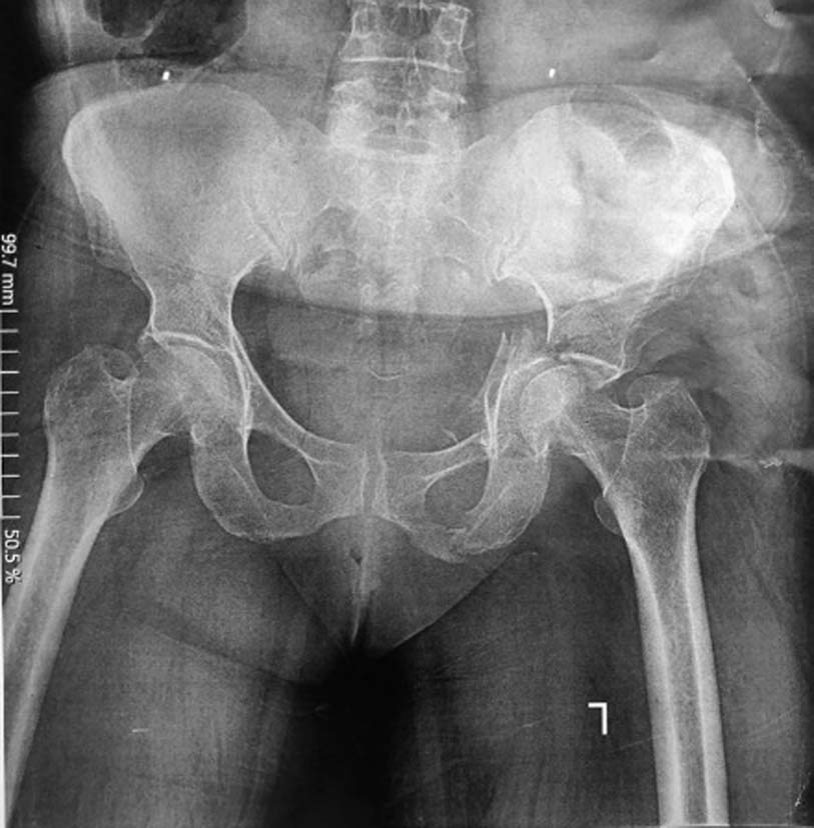
Plain pelvic radiograph before the fixation surgery (anteroposterior view).

A 57-year-old healthy woman was brought to the emergency department with a left acetabular fracture due to a slip and fall accident as she fell on the left side of her body. As seen in the anteroposterior view of the plain pelvic radiograph shown in Figure 1, she was admitted to the orthopedic ward with a both-column acetabular fracture diagnosis.

**Figure 2:**
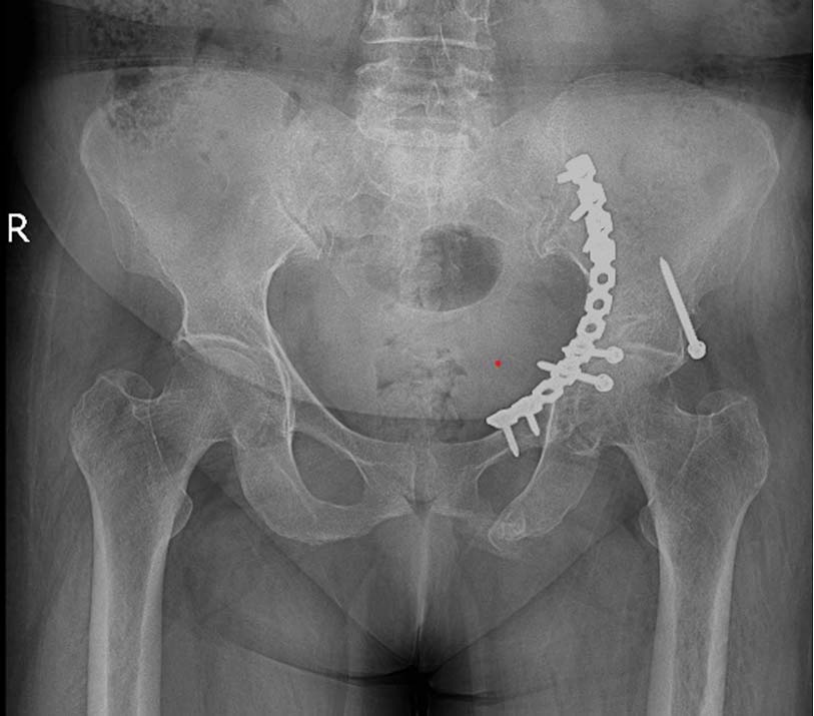
Plain pelvic radiograph after the surgery and fixation with plate and screws (anteroposterior view).

Neuromuscular and vascular examinations were routine, and the patient had no complaints except severe pain in the left inguinal region. After admission, the patient received antithrombotic medications for prophylaxis. Eventually, on the third day of admission, she underwent acetabular fixation surgery through the ilioinguinal approach with the implementation of a plate and screws for 105 min from incision to wound closure (Fig. 2).

**Figure 3:**
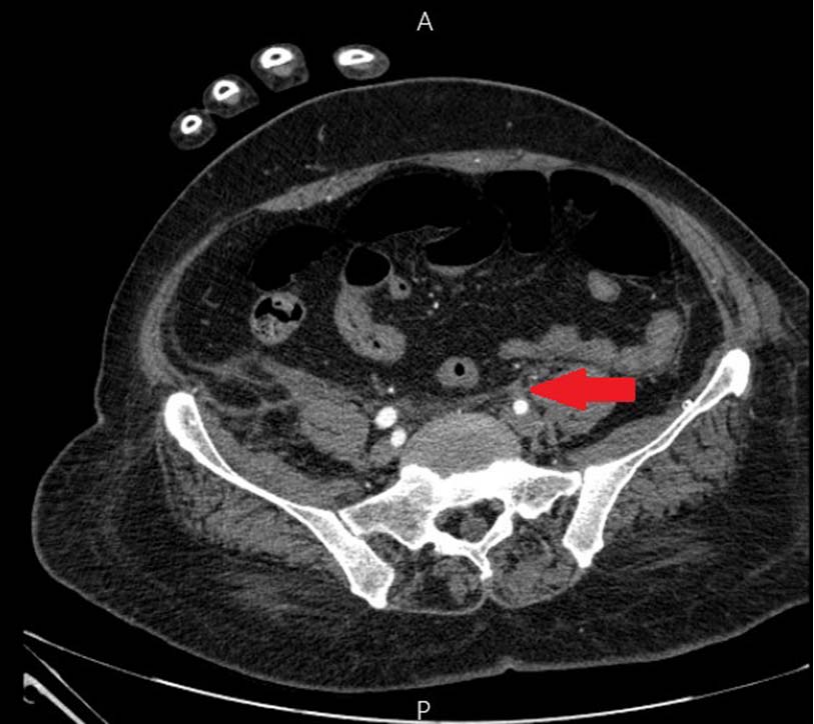
Axial pelvic computed tomography-angiogram showing occlusion in the left external iliac artery (the red arrow).

**Figure 4:**
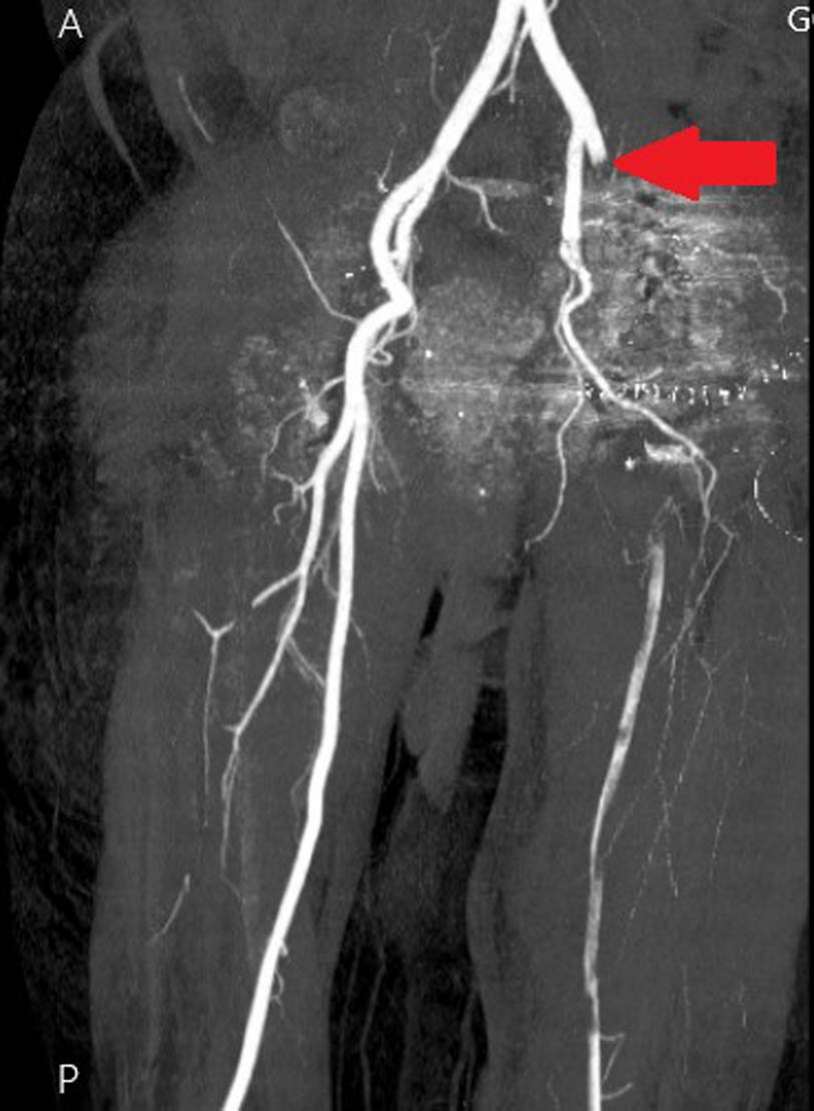
Coronal pelvic angiogram demonstrating left external iliac artery occlusion (the red arrow) after the bifurcation of the left common iliac artery.

During her convalescence in the hospital (4 h after surgery), she experienced coldness accompanied by skin pallor in the left leg. In addition, computed tomography angiography was done for the patient and revealed left external iliac artery thrombosis leading to surgical thrombectomy consequently (Figs 3, 4).

After therapy, the patient’s symptoms resolved, and she was discharged in good health. She was followed up for 15 months, and during this time, duplex ultrasonography was performed and demonstrated a typical flow pattern in the left leg arterial circulation.

## Discussion

Traumatic pelvic bone and acetabulum fractures account for ~2% of all fractures, with motor vehicle accidents and falls being the most common causes of injury^11^. The incidence of acetabular fractures in elderly patients due to traumatic injuries is increasing^12^. Nonoperative treatments have been associated with a high mortality rate, with 33% of patients with acetabular fractures reported to have died within one year and 84% of the deceased patients having received nonoperative treatments^11^.

Treating acetabular fractures presents numerous complications and difficulties, including associated major organ injuries, complex fracture types, and challenges in the operative approach for reduction^13^. These complications can create uncertainty regarding the preferred ilioinguinal approach for treating complex acetabular fractures^14^. However, most studies indicate that internal fixation of acetabular fractures leads to favorable outcomes in the majority of patients^3^.

The ilioinguinal approach provides a wide window to the anterior column of the acetabulum through an incision made from the symphysis pubis to the sacroiliac joint. Letourneau^4^ reported an 87% success rate in achieving perfect reduction using this approach for acetabular fractures.

Although the ilioinguinal approach is commonly used for managing fractures of the anterior column of the acetabulum, it carries a risk of arterial thrombosis. Previous literature suggests that this complication is primarily associated with tissue damage during fracture reduction and the mispositioning of devices or implants. Micro-injuries and stresses during the operation have also been proposed as potential causes of arterial thrombosis^9^.

A systematic review conducted by Kelly et al. in 2020^15^, which included multiple studies and a total of 8389 acetabular fractures, highlighted the need for high-quality data to accurately assess possible complications and outcomes.

Our article adheres to the SCARE criteria, ensuring comprehensive reporting of the case^16^.

## Conclusion

While the ilioinguinal approach for acetabular fixation may carry a minimal risk of rare complications such as arterial thrombosis, a thorough vascular examination and routine measurement of distal arterial pressure in the limbs after surgery can help prevent serious complications. Further research is necessary to better understand the incidence and management of complications associated with acetabular fractures.

## Limitation

Based on previous studies, external iliac artery thrombosis in the ilioinguinal approach seemed to be rare. However, this study showed a well-controlled manifestation of this life-threatening complication. Further comprehensive studies are necessary to investigate the prevalence and management of this complication.

## Ethical approval

Our study is a case report without any intervention on the patient, and informed consent has been obtained for publication.

## Consent

Written informed consent was obtained from the patients for the publication of this case report and accompanying images. A copy of the written consent is available for review by the Editor-in-Chief of this journal on request.

## Sources of funding

Not applicable.

## Author contribution

D.H.: idea, supervision, revising the manuscript, confirming final draft, and guaranteeing all details of the project; P.S.: data collection, literature review, writing the initial draft, revising the manuscript, confirming the final draft, and guaranteeing all details of the project; N.G.: data collection, revising the manuscript, confirming the final draft, and guaranteeing all details of the project; A.H.: revising the manuscript, confirming the final draft, and guaranteeing all details of the project; S.A.: idea, supervision, revising the manuscript, confirming final draft, and guaranteeing all details of the project.

## Conflicts of interest disclosure

The authors declare no conflicts of interest.

## Research registration unique identifying number (UIN)

Our article is a case report.

## Guarantor

Salman Azarsina.

## Data availability statement

Not applicable.

## Provenance and peer review

Not commissioned, externally peer-reviewed.
